# The newest COI molecular detection of Asian redtail catfish *Hemibagrus nemurus* (Valenciennes, 1840) in Progo River, Magelang, Central Java, Indonesia

**DOI:** 10.5455/javar.2022.i628

**Published:** 2022-11-18

**Authors:** Desyiamililia Yuanawati, Hutama Satriana Farizky, Muhammad Browijoyo Santanumurti, Mamdoh T. Jamal, Lalu M. Iqbal Sani, Hawis Madduppa, Putri Desi Wulan Sari

**Affiliations:** 1 Program Study of Aquaculture, Faculty of Fisheries and Marine, Universitas Airlangga, Surabaya, Indonesia; 2 Department of Aquaculture, Faculty of Fisheries and Marine, Universitas Airlangga, Surabaya, Indonesia; 3 Department of Marine Biology, Faculty of Marine Sciences, King Abdulaziz University, Jeddah, Kingdom of Saudi Arabia; 4 Oceanogen Environmental Biotechnology Laboklinikum, Cilendek Timur, Bogor, Indonesia

**Keywords:** DNA barcoding, Hemibagrus nemurus, Indonesia, Progo River

## Abstract

**Objective:**

This study describes the newest deoxyribonucleic acid (DNA) barcoding Asian redtail catfish (*Hemibagrus nemurus*) in the Progo River, Magelang, Central Java, Indonesia.

**Materials and Methods:**

Ten fish were caught in the Progo River, Magelang, Central Java, Indonesia. The polymerase chain reaction was the molecular diagnosis to detect the sequences of DNA of Cytochrome Oxidase I compared to National Center for Biotechnology Information data (GenBank).

**Results:**

The results showed that the percent identity was not 100% with *H. nemurus* data from other locations (GenBank), including Indonesia. The closest percent identity was *H. nemurus* from Java Island (Accession ID: MK312566.1) with 97.6% similarity. The genetic mutation that happened might be due to environmental change (pollution) in the Progo River recently.

**Conclusions:**

This study showed a genetic mutation in *H. nemurus* from Progo River may be caused by environmental change. Low pollution exposure levels may not be detrimental (lethal) to fish. However, it can affect fish fertility, which leads to population degradation (gene variation). Attention must be increased for fish survival in the future.

## Introduction

Indonesia being located in the tropics has a high diversity of flora and fauna, which is commonly referred to as “mega biodiversity.” There are at least 3,424 fish species with a total of 237 tribes in Indonesia [1]. These species variations can be affected by environmental conditions, namely temperature, pH, substrate, and even pollutants [2,3]. Many factors influence the high and low variation of fish species. One of the most crucial is environmental quality [4]. Biodiversity plays a very important role as a source of germplasm, ecosystem stability, and an economic source for locals. The Asian redtail catfish (*Hemibagrus nemurus*) is one of Indonesia’s freshwater endemic fish distributed in the islands of Java, Kalimantan, and Sumatra with high economic potential.

The Asian redtail catfish has become an economically important fish in several regions in Indonesia. This is due to the characteristics of Asian redtail catfish that have a larger size, high fecundity (30,000–70,000 eggs), are adaptive to artificial feed, have an enjoyable taste, and are popular in the community [5,6]. Asian redtail catfish also have a relatively expensive price, which ranges from IDR 75,000–100,000 or USD 5–7 per kg [5]. The Asian redtail catfish is one of the freshwater fish that has great potential in aquaculture. Currently, Asian redtail catfish have been successfully cultivated in various environments [5]. However, on the other hand, fish cultivation has not been widely practiced, and people still rely on catches from inland waters to consume fish. The Magelang Regency is one of the areas in Central Java (Indonesia) where the community makes Asian redtail catfish the most frequently consumed because it is pretty easy to find in rivers in that area.

In Magelang Regency, the Asian redtail catfish has a local name “Beong fish.” This fish inhabits one of the major rivers in Magelang Regency, namely Kali Progo, which flows from Mounts Sindoro, Sumbing, and Merapi. It is characterized by its reasonably swift current, which allows the distribution of Asian redtail catfish in this place. The Asian redtail catfish is a type of fish that only inhabits certain waters. This causes the natural stock to decrease along with the rise of fishing and water pollution. The Progo River has experienced a decrease in water quality to a light pollution level due to household and industrial activities around the river [2]. The dominant industry around Progo River is the tofu/tempeh production house. Although it is still in the category of light pollution, if this continues, it will be able to endanger the survival of aquatic organisms. Additionally, a previous study stated that life history characteristics (exposed broodstock) and habitat (environment quality) determine the genetic diversity patterns. This can also mean that environmental pollution can affect the genetics of organisms in the environment [7].

Therefore, it is necessary to identify genetic variations in Asian redtail catfish in the Progo River to determine the genetic changes that may occur due to decreased water quality caused by industrial and household pollution. These genetic variations are essential in short-term evaluations (individual fitness) and long-term evaluations such as survival in a population [8]. Genetic variation can be affected by predicting molecular markers such as mitochondrial deoxyribonucleic acid (DNA). One type of method that is most frequently used is DNA barcoding [9].

DNA barcoding is a genetic test method based on differences in the nucleotide sequence of genes with a standard of 655 base pairs (bp) using the Cytochrome Oxidase I (COI) gene so that it can be used to identify a species with a high degree of conformity as a key molecular marker that has certain effectiveness for tracking products [10]. Most of the time, this method is also used to tell the difference between species in the same genus or to figure out the taxonomy of species using short genetic markers derived from standard DNA genomes based on the amplification of short DNA found in the mitochondrial genome [11].

Based on current conditions in the Progo River and the urgency of identification of Asian redtail catfish, it is necessary to identify genetic variations in Asian redtail catfish from the Progo River to determine the genetic changes that may occur due to decreased water quality caused by pollution. This study also identified the relationship between fish of the same genus through DNA barcoding data previously reported at National Center for Biotechnology Information (NCBI) GenBank.

## Materials and Methods

### Sample collection

**Figure 1.**
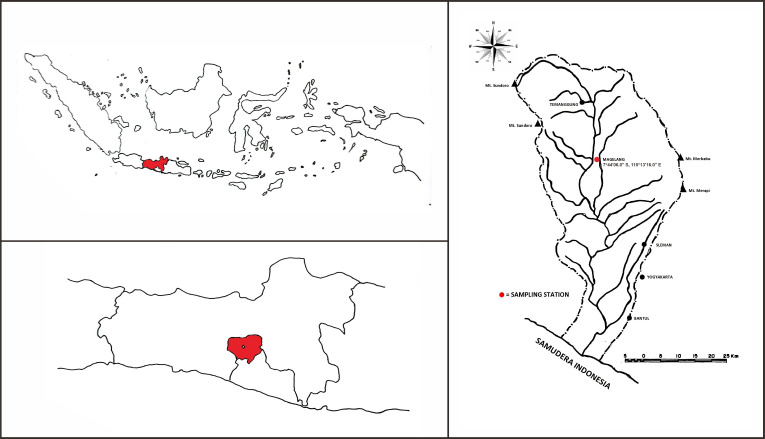
Geographical map of the sampling stations. A. Indonesia map (Red Color: Central Java), B. Central Java: (Red Color: Magelang), C. Hydrology of the Progo River Basin, Magelang, Central Java, Indonesia.

**Figure 2.**
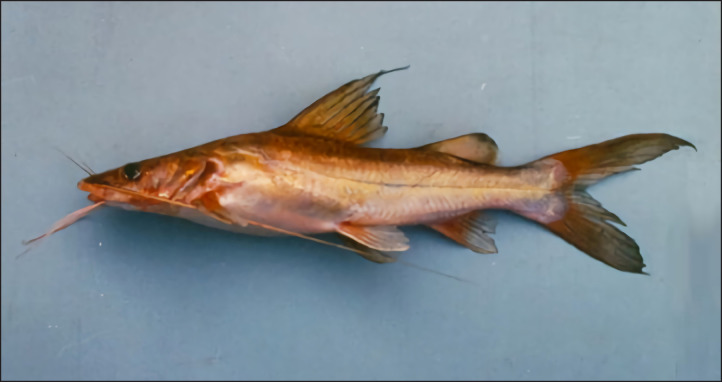
Asian redtail catfish (*H. nemurus*) (Baird [14]).

Approximately 10 samples were collected on September 12, 2021, from the Progo River, Magelang, Central Java (7°44’06.0” S, 110°13’16.0” E) (Fig. 1). The tissue samples were obtained from the dorsal fin of an Asian redtail catfish (*H. nemurus*) [Fig. 2]. The sample surface was cleaned with aquades, inserted into a 2 ml Cryotube that already contained 96% liquid ethanol, and labeled with the sampling code “21MGL_BNG_01” for the first sample [12]. After that, the sample was ready to proceed to the DNA extraction stage.

### Molecular detection

DNA extraction was conducted using Geneaid gSYNC kit extraction that adheres to the manufacturer’s procedure. To visualize and check the DNA extraction results, 2% gel electrophoresis was used. polymerase chain reaction (PCR) methods used a primer Fish F1 (5’-TCA ACC AAC CAC AAA GAC ATT GGC AC-3’) and Fish R1 (5’-TAG ACT TCT GGG TGG CCA AAG AAT CA3’) [13].

In this process, the amplification of PCR was conducted in 40 cycles. The pre-denaturation stage was carried out at a temperature of 94°C for 2 min, denaturation at 94°C for 45 sec, annealing at a temperature of 45°C for 45 sec, extension at 72°C for 1.5 min, and final extension at 72°C for 10 min. This cycle is repeated 40 times. After the DNA amplification process, 2% gel electrophoresis was used to visualize and check the DNA extraction result. Electrophoresis was then conducted by transferring 4 μl amplified DNA into an agarose well, dissolved in the TAE buffer, then stained using GelRed™ and electrophoresed at 110 V for 20 min. After that, the electrophoresis results can be observed with a UV transilluminator. After these several stages, sequencing is carried out, which is the final DNA barcoding stage. The electrophoresis process used agarose gel for DNA and polyacrylamide for protein, while Ethidium Bromide was used for visualization [9].

The sequence was obtained from NCBI GenBank, then analyzed using the NCBI Basic Local Alignment Search Tools (BLAST). The selected COI sequences will be stored using the FASTA format for further sequence alignment using the NCBI BLAST [15]. The phylogenetic tree was created based on the Fast Minimum Evolution analysis tree method. Sequence alignment was conducted by editing and alignment building via ClustalW in the MEGA X application.

## Results

### Sample sequences

The sequence results came from the best sample of Asian redtail catfish (*H. nemurus*) taken from the Progo River, Magelang, Central Java Province. The results showed that sample 21MGL_BNG from Progo River was an *H. nemurus* species because it had a high Percentage Identity value of 99.6% with *H. nemurus* from Palembang, Musi River, and Lampung in Indonesia. Detailed information on the sample sequence after BLAST based on the DNA molecule is represented in Table 1.

**Figure 3.**
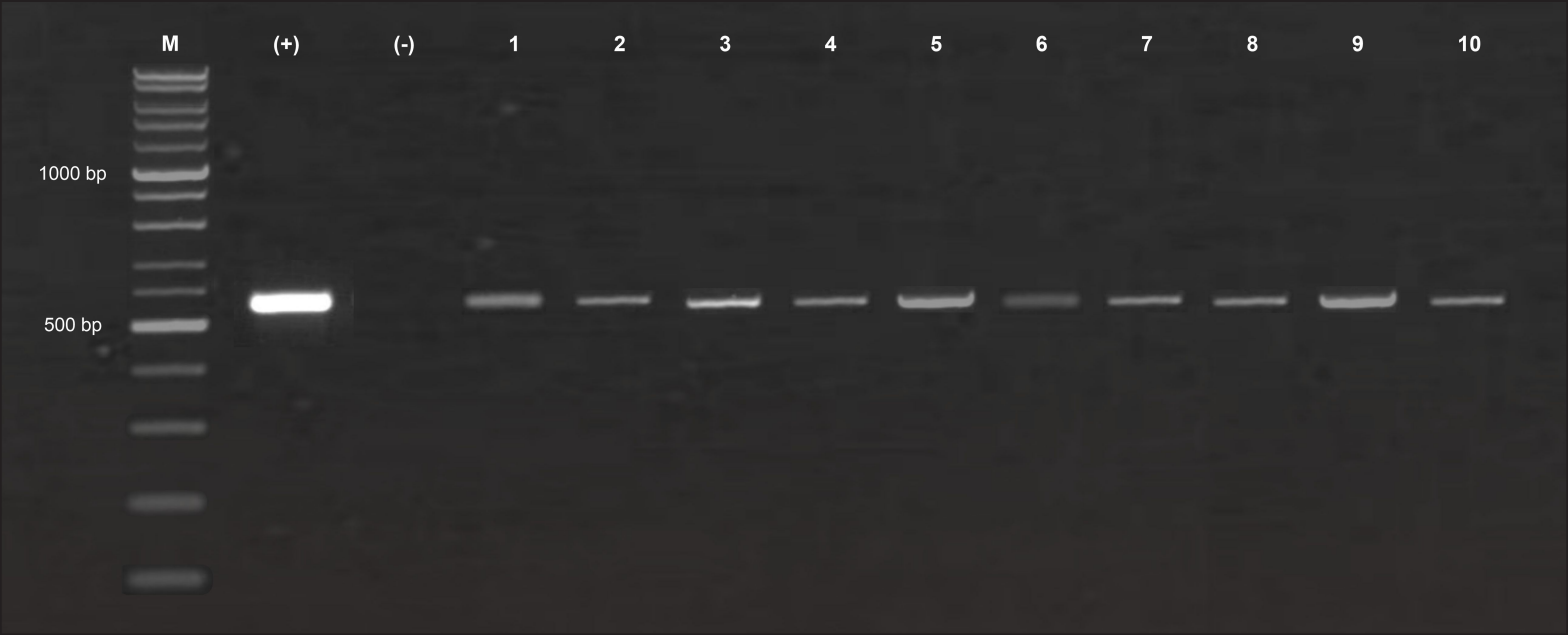
*H. nemurus* samples product bands from PCR amplification on 2% (w/v) agarose gels under UV light. M: 1 kb marker, +/− (positive/negative) control.

Similar to the results of Table 1, the results of the analysis of sample sequences after BLAST based on RNA molecules also stated that the sample 21MGL_BNG from Progo River was an *H. mumurus* species because it had a high Percentage Identity value of 99.6% with *H. nemurus* from Palembang, Musi River, and Lampung in Indonesia. Detailed information on the sample sequence after BLAST based on the RNA molecule is represented in Table 2. Overall, the 10 samples we tested were positive for *H. nemurus*, indicated by the presence of bands at 563 bp (Fig. 3). This could confirm the correctness of the species naming in the samples we studied.

**Table 1.** Identification result (Query Coverage and Percent Identity) of Asian redtail catfish (*H. nemurus*) sample in the Progo River compared with the same genus using BLAST (DNA Molecule Type).

Species	Location	Accession ID*	Query coverage (%)	Percent identity (%)
			21MGL_BNG (*H. nemurus* from Progo River)	
*Hemibagrus nemurus*	Palembang*,* ***Indonesia***	KM213068.1	100	99.6
*Hemibagrus nemurus*	Musi River*,* ***Indonesia***	MG521912.1	100	99.6
*Hemibagrus nemurus*	Lampung*,* ***Indonesia***	MN243484.1	100	99.6
*Hemibagrus nemurus*	Java, ***Indonesia***	MK312566.1	82	97.6
*Hemibagrus nemurus*	Sabah, ***Malaysia***	JN646095.1	100	94.5
*Hemibagrus nemurus*	***Thailand***	JQ289148.1	95	93.7
*Hemibagrus nemurus*	Java and Bali, ***Indonesia***	KU692545.1	100	87.6
*Hemibagrus nemurus*	***China***	JN020074.1	100	85.4
*Hemibagrus planiceps*	Pelus River, Purwokerto, Central Java, ***Indonesia***	KU692546.1	100	87.7
*Hemibagrus menoda*	***Bangladesh***: Mymensingh, Kishoreganj, Itna Haor, Itna, Kishoreganj	MK572258.1	100	87.6
*Hemibagrus fortis*	Sabah, ***Malaysia***	KT799807.1	100	87.4
*Hemibagrus maydelli*	***India***: Northern Western Ghats	KX946649.1	100	86.7
*Hemibagrus macropterus*	***China***: Jiangsu, Zhenjiang	MF122325.1	100	86.5
*Hemibagrus guttatus*	***China***: Guangdong, Zhaoqing, Deqing	MT884560.1	99	86.3
*Hemibagrus wycoides*	***Thailand***: Mekong River Basin	EU490862.1	100	85.1

* These data were obtained from BLAST analysis on GenBank NCBI.

**Table 2.** The result of identification (Query Coverage and Percent Identity) from Asian redtail catfish (*H. nemurus*) sample in the Progo River compared with the same genus using BLAST (RNA Molecule Type).

Species	Location	Accession ID*	Query coverage (%)	Percent identity (%)
			21MGL_BNG (*H. nemurus* from Progo River)	
*Hemibagrus nemurus*	Palembang*,* ***Indonesia***	KM213068.1	100	99.6
*Hemibagrus nemurus*	Musi River*,* ***Indonesia***	MG521912.1	100	99.6
*Hemibagrus nemurus*	Lampung*,* ***Indonesia***	MN243484.1	100	99.5
*Hemibagrus nemurus*	Java, ***Indonesia***	MK312566.1	82	97.6
*Hemibagrus nemurus*	Sabah, ***Malaysia***	JN646095.1	100	94.5
*Hemibagrus nemurus*	***Thailand***	JQ289148.1	95	93.7
*Hemibagrus nemurus*	***China***	KM454860.1	100	97.7
*Hemibagrus nemurus*	Selangor,*** Malaysia***	KT001039.1	98	88.3
*Hemibagrus planiceps*	Pelus River, Purwokerto, Central Java, ***Indonesia***	KU692546.1	100	87.7
*Hemibagrus menoda*	***Bangladesh*: **Mymensingh, Kishoreganj, Itna Haor, Itna, Kishoreganj	MK572258.1	100	87.6
*Hemibagrus fortis*	Sabah, ***Malaysia***	KT799807.1	100	87.4
*Hemibagrus maydelli*	***India***: Northern Western Ghats	KX946649.1	100	86.7
*Hemibagrus macropterus*	***China***: Jiangsu, Zhenjiang	MF122325.1	100	86.5
*Hemibagrus guttatus*	***China***: Guangdong, Zhaoqing, Deqing	MT884560.1	99	86.3
*Hemibagrus wyckioides*	***Thailand***: Mekong River Basin	EU490862.1	100	85.1

* These data were obtained from BLAST analysis on GenBank NCBI.

### Phylogenetic analysis

The BLAST results based on the DNA molecule of the *H. nemurus* specimen are also shown in Table 1. Sixteen sequences [15 sequences from Bangladesh, China, India, Indonesia (Musi River; Pelus River; Lampung; Java; Bali), Malaysia, Thailand, and 1 sequence from this study] of mitochondrial DNA of the COI gene (563 bp length) were obtained after modification and alignment.

**Figure 4.**
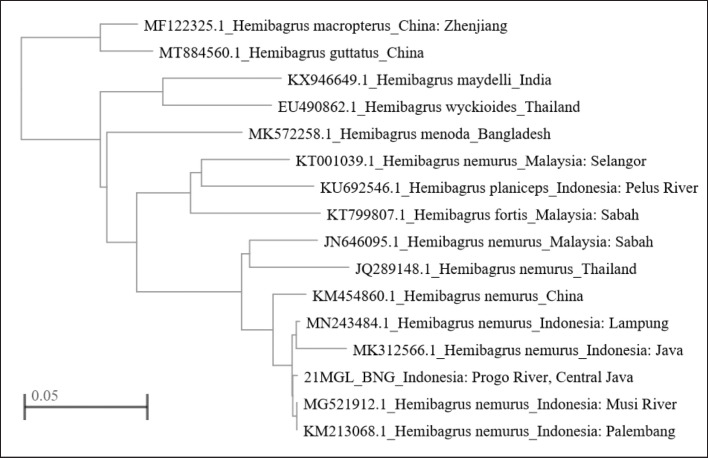
Fast Minimum Evolution tree method using sample of 21MGL_BNG (Progo River) of with the same Genus sequences based on DNA Molecule Type (NCBI).

The phylogenic tree of this genus showed a monophyletic group in which four lineages were described. The first clade consisted of *H. macropterus* and *H. guttatus* from China*.* The second clade consisted of *H. maydelli* from India and *H. wyckioides* from Thailand. *H. nemurus* from the Progo River subclade was placed in the sister clade of *H. nemurus* from Lampung (Accession ID: MN243484.1), *H. nemurus* from Palembang (Accession ID: KM213068.1), *H. nemurus* from Musi River (Accession ID: MG521912.1), and *H. nemurus* from Java (Accession ID:MK312566.1) (Fig. 4).

The BLAST results based on the RNA molecule of the *H. nemurus* specimen are also shown in Table 2. As with BLAST results based on DNA molecule type, 15 sequences were obtained from the GenBank, and one sequence from the present mitochondrial RNA study of the COI gene (563 bp) was obtained after modification and alignment.

**Figure 5.**
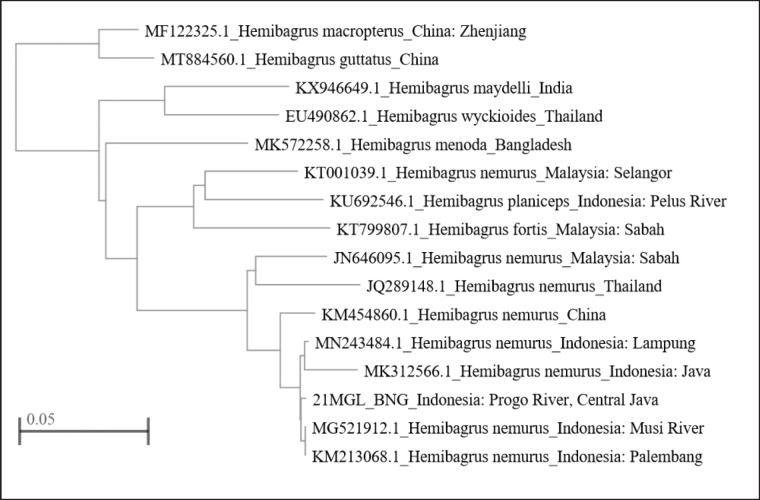
Fast Minimum Evolution tree method using sample of 21MGL_BNG (Progo River) of with the same Genus sequences based on RNA Molecule Type (NCBI).

The phylogenic tree based on RNA molecule type has the same results as the results of the phylogenic tree analysis based on DNA molecule type. *Hemibagrus nemurus* from Progo River was placed in one subclade with *H. nemurus* from Lampung (Accession ID: MN243484.1), *H. nemurus* from Palembang (Accession ID: KM213068.1), *H. nemurus* from Musi River (Accession ID: MG521912.1), and *H. nemurus* from Java (Accession ID: MK312566.1) (Fig. 5).

## Discussion

### The importance of COI gene

In 2003, DNA barcoding technology was proposed, which used the mitochondrial cytochrome c oxidase subunit I (COI) gene sequence for species identification barcoding to be able to barcode all species for identification and classification. The COI intraspecific diversity gene in animals was found to be significantly lower than the interspecific diversity. The COI gene barcoding was effective for classifying and identifying vertebrates and invertebrates, so it has been widely used in various fisheries’ biological groups [16–19]. This is what makes COI genes very important in the identification and classification of biological groups.

COI-based DNA barcoding has demonstrated its potency as a molecular tool for identifying new species [20]. There are many COI gene-based relevant publications in fisheries organism identification, namely, Siberian sturgeon (*Acipenser baerii*) [21], Narrow worm eel (*Scolecenchelys macroptera*) [22], Giant catfish (*Netuma thalassina*) [22], Striped eel catfish (*Plotosus lineatus*) [22], and Bombay duck (*Harpadon nehereus*) [22]. DNA barcoding has also proven to be essential in verifying the validity of existing taxonomy and edifying examples of improper synonyms and unobserved taxa [23]. As validated in marine fish [20,24], COI regions can differentiate closely related species between different phyla. So, DNA barcoding has the potential to tell the difference between organisms that do not have any unique physical traits [25]. This makes it useful for monitoring biodiversity, taxonomy, ecology, and conservation research [26].

The mitochondrial cytochrome oxidase subunit 1 (COI) gene is one of the most prevalent markers for systematic molecular application. These gene fragments are frequently used to infer phylogenies, specifically near the 5’-end region used by the DNA Barcoding Consortium. The DNA barcoding database is filled with an ever-growing number of sequences, thus the urgent need to understand this gene evolution and its evolutionary relationship among species; the informative potential is also necessary to be analyzed for the phylogenetic inference genes used by each group [27].

The main benefits that will be obtained when using DNA barcoding (COI gene) technology compared to traditional classification methods to classify and identify vertebrates and invertebrates include the following: 1) Because some organisms have high external morphological similarity, we find it difficult to distinguish them from one another based solely on their external morphology. DNA barcoding technology (COI gene) can help differentiate between organisms (species) more accurately. 2) External morphological differences can be different in each stage of development. However, individuals in different stages of development can still be accurately identified with DNA barcoding (COI gene) technology. The cryptic species’ intelligibility can be discovered using DNA barcode technology (COI gene). Cryptic species are two or more morphologically similar but genetically different species. Due to morphological similarities, cryptic species are often identified and classified as the same species in the conventional system. DNA barcode technology (COI gene) can show how far apart these species are in molecular evolution, shedding light on species that were previously hard to understand [22].

The COI gene has a key role in a global effort to document biodiversity and continues to be a chosen gene in phylogenetic and phylogeographic studies [28]. DNA barcode analysis attempts to identify the boundaries that delineate species, corresponding to the divergence between the nearest neighbors within a group [20,29].

### Molecular identification

From the results of this study, 563 bp were obtained from the sample 21MGL_BNG. The results of DNA barcoding (563 bp) were then compared with the existing database at NCBI (Table 1). There are pretty exciting facts after the results, namely the genetic variation (nucleotides) from samples of 21MGL_BNG to data on sequences of Asian redtail catfish (*H. nemurus*) caught from various locations in NCBI GenBank data. First of all, after our BLAST analysis, we know that the 21MGL_BNG fish sample we tested is of the *H. nemurus* species. There is an interesting fact from the information from the BLAST analysis that we have carried out, namely about the percentage identity of the 21MGL_BNG fish sample to the *H. nemurus* sequence data from other locations, none of which has a 100% percent identity value. Even with the *H. nemurus* sample from Java Island (Accession ID: MK312566.1), it only has a similarity of 97.6%. Compared with *H. nemurus* taken from the islands of Java and Bali (Accession ID: KU692545.1), it has a lower identity percentage value of 87.6%, which can be seen in (Table 2). Before starting, it is essential to know that the percent identity value is the highest match between the query sequence and the aligned database (NCBI) sequence [30].

The findings advanced the doubt that there has been a genetic mutation in the 21MGL_BNG fish sample, as fish have been compared with fish of the same species and exact location, namely the Java island. The second reason this allegation arose was also due to the previous research, which stated that the water quality in the Progo River was in a “lightly polluted” status. This happened on the Progo River, Magelang, Central Java [2]. In line with this, low pollution exposure may not be lethal to fish [31]. However, it can affect fish fertility, which leads to population degradation (gene variation). In addition, a previous study also revealed that pollution might affect regional genetic diversity, aside from other factors, namely fishing activities, adsorption mechanisms, and seasonal influence [32].

Fishing activities allow natural fish populations to be less stressed and lose genetic variability [33]. In Magelang, fishing activities are also massive and are estimated to affect the biodiversity of fish in the Progo River, including the availability of Asian redtail catfish [34-35]. Furthermore, the season will also play a significant role in influencing the genetic diversity of a fish population, and fish that cannot adapt (mutate) to seasonal changes will become extinct [36]. Martinez et al. [7] in the conclusion of their research also say that both life history characteristics (exposed broodstock) and habitat (environmental quality) affect the genetic diversity patterns in fishes.

In addition, the Query Coverage in the 21MGL_BNG sample compared to the *H. nemurus* sample from Java Island (Accession ID: MK312566.1) only has 82%, which can be seen in (Table 2). We should have guessed that it is very likely that with almost the same pick-up location, namely on the island of Java, it will have a high Query Coverage score (100%). After we studied the sequencing data from the *H. nemurus* sample from Java Island (Accession ID: MK312566.1), it was found that in the data collected in 2012–2013, the nucleotide sequence of genes was worth 456 bp. This causes the score The Query Coverage is 82% because the nucleotide sequence of genes from the 21MGL_BNG sample is 563 bp.

This mutation type is called a “frameshift mutation.” In this case, it is suspected that a nucleotide gene insertion process occurred despite the many factors that can cause gene mutations in aquatic organisms. In this study, it is assumed that this mutation occurs due to pollutants affecting the water quality. It is not surprising that mutations can occur because the sequence data (Accession ID: MK312566.1) was collected in 2012–2013, which is almost 10 years. We already have the latest sequence data (*H. nemurus*) collected at the end of 2021 at the sampling site of the Progo River, Magelang, Central Java. The allegation of mutations caused by pollution is also getting stronger after previous research has found that the waters in the Progo River have been partially polluted [2].

Based on these phylogenetic results, the *H. nemurus* species from the Progo River sample (21MGL_BNG) are related to other *H.* species. From the phylogenetic tree, *H. nemurus* from the Progo River sample has a relationship with H. *nemurus* from Lampung (Accession ID: MN243484.1), *H. nemurus* from Palembang (Accession ID: KM213068.1), *H. nemurus* from Musi River (Accession ID: MG521912.1), and *H. nemurus* from Java (Accession ID: MK312566.1), which can be proven from the lines in the subclade (Figs. 4 and 5). *H. macropterus* has a close relationship with *H. guttatus* seen from their placement in one clade, while *H. maydelli* also has a close relationship with *H. nemurus* from China and *H. wyckioides* from Thailand in the same clade [37]. The COI gene sequences can be used to discern *H.* from other Bagridae from Bangladesh, China, India, Malaysia, and Thailand. Molecular species authentication techniques have been confirmed to create a potentially rapid and accurate assessment of proper labeling [38].

A phylogenetic tree describes the evolutionary lineage of a species, organism, or common ancestor [39]. Based on the phylogenetic tree made, it can be seen the genetic relationship between species in one population and between populations. Phylogenetics can also be used to organize biological diversity knowledge for structural classification and to provide event insight during evolution [40,41].

A DNA molecule consists of two long polynucleotide chains, each composed of four nucleotide subunit types. Each of these chains is known as a DNA chain or a DNA strand. The hydrogen bonds between the base portions of the nucleotides hold the two chains together. Nucleotides are composed of five-carbon sugars attached to one or more phosphate groups and a nitrogen-containing base. In DNA nucleotides, the deoxyribose sugar is attached to a single phosphate group (hence the name DNA), and the base may be either adenine (A), cytosine (C), guanine (G), or thymine (T). Genetic information is carried in the linear sequence of DNA nucleotides. Each DNA molecule is a double helix of two complementary nucleotide strands held together by G-C and A-T base pair hydrogen bonds. Genetic information duplication occurs using one DNA strand as a template for a complementary strand formation. The DNA of organism stores the instructions for making all of its proteins.

In this study, we used BLAST analysis not only for molecular DNA but also for molecular RNA (Table 2). After the results of the BLAST analysis (DNA and RNA molecule types) were obtained, we compared them and found that the results of the BLAST analysis between the two (DNA and RNA molecule types) were the same. In line with this, the results of the Fast Minimum Evolution tree method analysis on both types of molecules (DNA and RNA) show that the 21MGL_BNG samples occupy the same position and sub-clusters in the phylogenetic tree. RNA (ribonucleic acid) is similar to DNA (DNA). However, it is a strong and lively member of the nucleic acid family. The two main differences between RNA and DNA are that 1) RNA differs from DNA by one nucleotide and 2) RNA incorporates a single strand (one nucleotide) rather than a double helix [42]. The base may be either adenine (A), cytosine (C), guanine (G), or uracil (U).

DNA and RNA are the most essential molecules in cell biology, chargeable for storage and analyzing genetic records, which are the basis of all existence. DNA encrypts all genetic records and is the prototype that creates all organic existence. RNA functions as the decoding reader. There are three RNA types: 1) mRNA—copies genetic code fragments through a process called transcription and transports them to ribosomes (cellular factories that enable protein production from this code), 2) tRNA—engaged in bringing amino acids, basic protein-building blocks, to these protein factories in response to the coded instructions received by mRNA; this protein building is called translation, and 3) rRNA—a ribosome component factory required for protein production [42].

## Conclusion

In conclusion, we obtained comparative data on Query Coverage and Percent Identity based on the types of DNA and RNA molecules in samples of Asian redtail catfish from Java, Indonesia. The sample identity percentage of our Asian redtail catfish is not 100% the same as the data of *H. nemurus* (Accession ID: MK312566.1) from the exact location, Java Island, in 2012–2013 according to the GenBank database (97.6%). This phenomenon is most likely caused by genetic mutations due to environmental changes (pollution) in the Progo River recently. To avoid the bad effects of pollution on the Progo River, it is essential to do more research on the topic.

## List of Abbreviations

COI I, Cytochrome oxidase I; PCR, Polymerase chain reaction; TAE, Tris-acetate-EDTA; DNA, Deoxyribonucleic acid; NCBI, National Center for Biotechnology Information; BLAST, Basic Local Alignment Search Tools.
